# Axial and frontal X-ray fluoroscopy technique of the sustentaculum tali can improve the accuracy of sustentacular screw placement

**DOI:** 10.1186/s12880-022-00898-z

**Published:** 2022-09-29

**Authors:** Jian-Ning Sun, Ai-Xiang Zhu, Ce Shi, Bei Zhang, Guang-Sheng Tang, De-Guang Wang, Wang Bing

**Affiliations:** 1 Department of Orthopedics, Suqian Hospital of Nanjing Drum Tower Hospital Group, Suqian, China; 2 Department of Orthopedics, The Affiliated Suqian Hospital of Xuzhou Medical University, Suqian, China; 3 Kangda College of Nanjing Medical University, Nanjing, China; 4 Xuzhou Medical University, xuzhou, China

**Keywords:** Sustentaculum tali, Axial and frontal, Calcaneal fracture, Fluoroscopy technique, Screw placement

## Abstract

**Introduction:**

Calcaneal fractures, especially those involving the articular surface, should be anatomically reduced as much as possible. Fixing the fracture by placing a screw into the sustentaculum tali from the lateral side of the calcaneus is generally considered to be the key to successful surgery. However, due to the limited visibility during surgery, it is not easy to place screws into the sustentaculum tali accurately. The purpose of this study was to explore a new fluoroscopy method for the sustentaculum tali and verify the value of this method in improving screw placement accuracy.

**Methods:**

In this study, a total of 42 human foot and ankle specimens were dissected and measured. The shape and position of the sustentaculum tali were observed, and the influence of adjacent bones on imaging findings was analysed. The axial and frontal X-ray fluoroscopy method to view the sustentaculum tali was formulated, and the appropriate projection angle through anatomical and image measurements was explored. Thirty specimens were randomly selected for screw placement, and the direction of the screw was dynamically adjusted under the new imaging method. The success rate of sustentacular screw placement was evaluated.

**Results:**

The anteversion angles of the sustentaculum tali were 30.81 ± 2.21° and 30.68 ± 2.86° by anatomical and imaging measurements, respectively. There was no statistically significant difference in the anteversion angle between the two measurement methods. Harris heel views should be obtained at 30° to identify the sustentaculum tali on axial X-ray images. Frontal X-ray imaging was performed perpendicular to this projection angle. Through frontal and axial X-ray imaging, the position and shape of the sustentaculum tali can be clearly observed, and these factors are seldom affected by adjacent bones. Under the new fluoroscopy method, the screws were placed from the anterior region of the lateral wall of the calcaneus to the sustentaculum tali. A total of 60 screws were placed in the 30 specimens; of these, 54 screws were in good position, 2 screws penetrated the cortical bone, and 4 screws did not enter the sustentaculum tali. The success rate of sustentacular screw placement was 90% (54/60).

**Conclusions:**

Axial and frontal X-ray images of the sustentaculum tali can clearly show the shape of the structure, which improves sustentacular screw placement accuracy.

## Introduction

The calcaneus is the most vulnerable tarsal bone and is mostly damaged by falls from a height and traffic accidents [1]. It has been reported that calcaneal fractures account for approximately 65% of foot fractures, and 75% of calcaneal fractures involve the articular surface of the calcaneus and talus [2]. Open reduction and internal fixation are effective methods for the treatment of calcaneal fractures, but the irregular shape of the calcaneus and the changes in bone shape after compression have been difficult obstacles to overcome in fracture reduction [3, 4]. Therefore, reliable intraoperative reference points and fixed points are keys to successful surgery [5].

As the only bony eminence support structure on the medial side of the calcaneus, the sustentaculum tali is rarely displaced in calcaneal fractures and is considered an ideal reference point for the reduction of calcaneal fractures [6]. The sustentaculum tali is rich in cortical bone; in addition, the dense trabecular bone continues with the trabecular bone in the anterior part of the calcaneus and the colliculus, which also provides a stable mechanical basis for the maintenance of screw fixation [7]. A number of studies have confirmed the reliability and effectiveness of sustentacular screw fixation in operations to treat calcaneal fractures [8–10].

However, the sustentaculum tali is located on the inside of the calcaneus; it is small in appearance and obscured by adjacent bones. Coupled with the limitation of the exposure range of the lateral incision for calcaneal fractures, it is difficult to precisely insert screws into the sustentaculum tali [11]. Experienced surgeons can dynamically observe and adjust the direction and depth of screw insertion through intraoperative X-ray fluoroscopy, but approximately 40% of surgeons are still dissatisfied with screw placement [12, 13]. Therefore, a precise and convenient X-ray fluoroscopy method for sustentaculum tali should be developed and promoted.

The purpose of this study was as follows: (1) The anatomical and imaging measurements of foot and ankle specimens were used to create axial and frontal X-ray fluoroscopy techniques for sustentaculum tali. (2) This new fluoroscopy technique was applied to place sustentacular screws in foot and ankle specimens, and its feasibility, reliability and effectiveness were evaluated. We hypothesized that this new fluoroscopy method would help improve the accuracy of sustentacular screw placement.

## Methods

After approval by the ethics committee, we performed dissection and measurements on 42 foot and ankle specimens. All specimens were from the Anatomy Department of Xuzhou Medical University. The specimens were from different individuals, regardless of age, sex and fractured side. The specimen included the middle and lower parts of the calf, ankle and foot, and the calf length was not less than 15 cm. In the process of specimen selection, we excluded individuals with large differences to make the obtained research data more suitable for the majority of the population and more clinically useful. Specimen inclusion criteria were as follows: (1) Chinese adult corpse specimen and (2) body length ≥ 150 cm and foot length ≥ 18 cm. Exclusion criteria were as follows: (1) congenital ankle dysplasia; (2) foot and ankle rheumatoid disease, severe osteoarthritis and other bone deformity diseases; and (3) foot and ankle tumour, bone tuberculosis and chronic osteomyelitis and other diseases. The specimen was fixed in the functional position of the ankle joint with a homemade fixation frame. An anatomy professor dissected the specimen, took pictures and marked the measurement line.

**Fig. 1.**
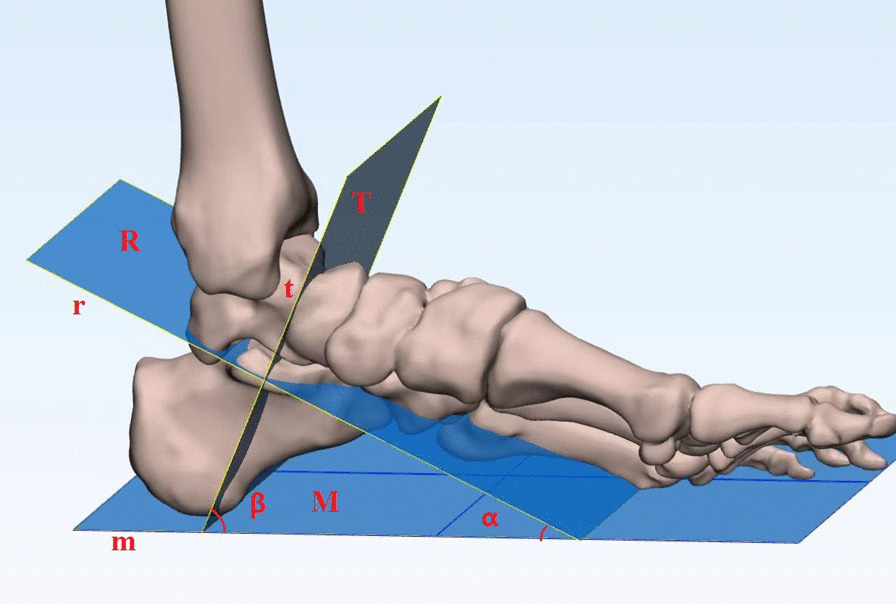
3D image of the medial ankle joint. M: plantar plane; R: coronal plane of long axis of the sustentaculum tali; T: short axis of the sustentaculum tali in the coronal plane; α: anteversion angle of the sustentaculum tali, also the axial projection angle of the sustentaculum tali; β: frontal projection angle of the sustentaculum tali

The sustentaculum tali was divided into three equal parts from the anterior to the posterior regions according to the body direction. The line connecting the midpoints of the three bisectors is the direction of the long axis of the sustentaculum tali. The long axis of the sustentaculum tali is defined as the straight line “r” along the sagittal direction of the sustentaculum tali body. The plane “R” is the coronal plane of the long axis of the sustentaculum tali. The short axis of sustentaculum tali is defined as the straight line “t” that passes through the midpoint of the long axis of the sustentaculum tali and perpendicular to the “R” plane. The plane “T” is the short axis of the sustentaculum tali coronal plane and perpendicular to the “R” plane. The plantar plane “M” is defined as the base surface of the specimen measurement. The angle “α” between the “R” and “M” planes is the anteversion angle of the sustentaculum tali, that is, the sustentaculum tali axial projection angle. The angle “β” between the “T” plane and the “M” plane is the sustentaculum tali frontal projection angle (Fig. 1).

### Specimen image

**Fig. 2:**
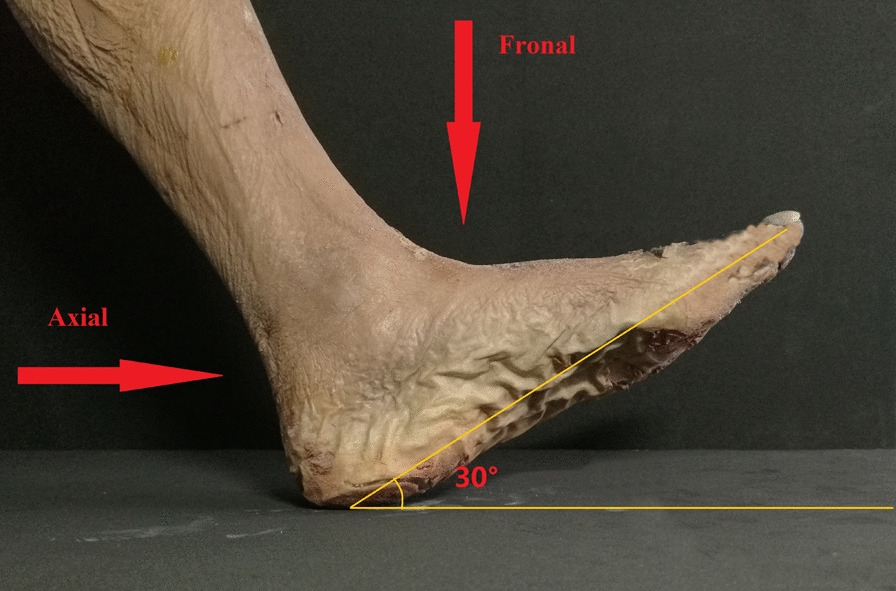
Schematic diagram of axial and frontal X-ray fluoroscopy of the sustentaculum tali

The specimen was fixed in the functional position. Each specimen was scanned by a 64-row 128-layer MSCT scanner with a pixel size of 0.3 mm from Siemens, Germany. The scan range included 10 cm of the lower leg, ankle joint and foot. The acquired image data were imported into Syngo MMWP VE36A workstation software for 3D reconstruction. According to the measurement needs, combinations of appropriate two-dimensional and three-dimensional reconstruction images were used. The positional relationships among the talus, medial malleolus, navicular bone and sustentaculum tali were analysed along with their influence on the anteroposterior and axial projection of sustentaculum tali. A complete set of PHILIPS Digital Diagnos X-ray camera system equipment was used to take axial and frontal X-ray images of the ankle joint anteroposterior, lateral, calcaneal axial, sustentaculum tali of each specimen (Fig. 2).

### Specimen dissection

**Fig. 3:**
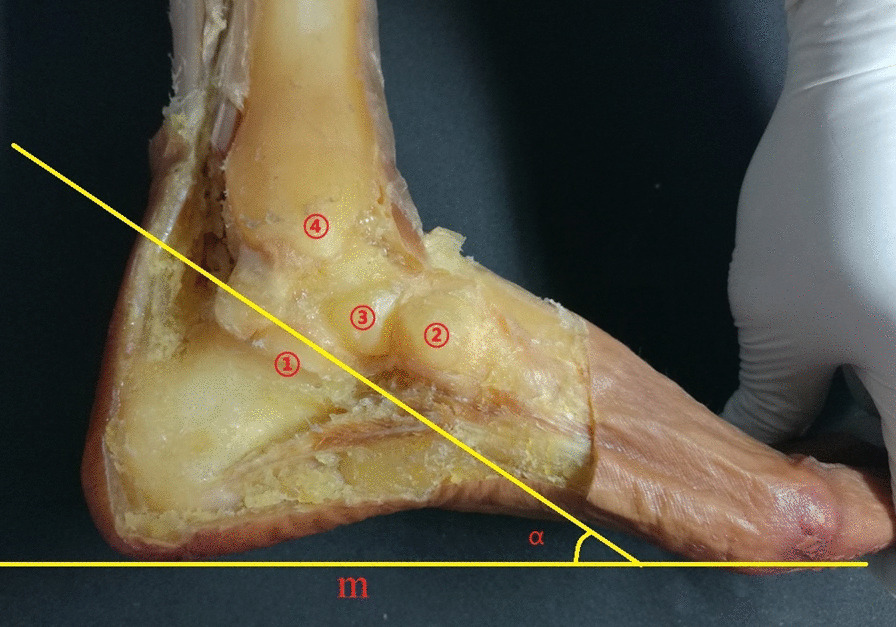
Anatomy of the medial ankle. ①: sustentaculum tali; ②: navicular bone; ③: posterior talus; ④: medial malleolus

**Fig. 4:**
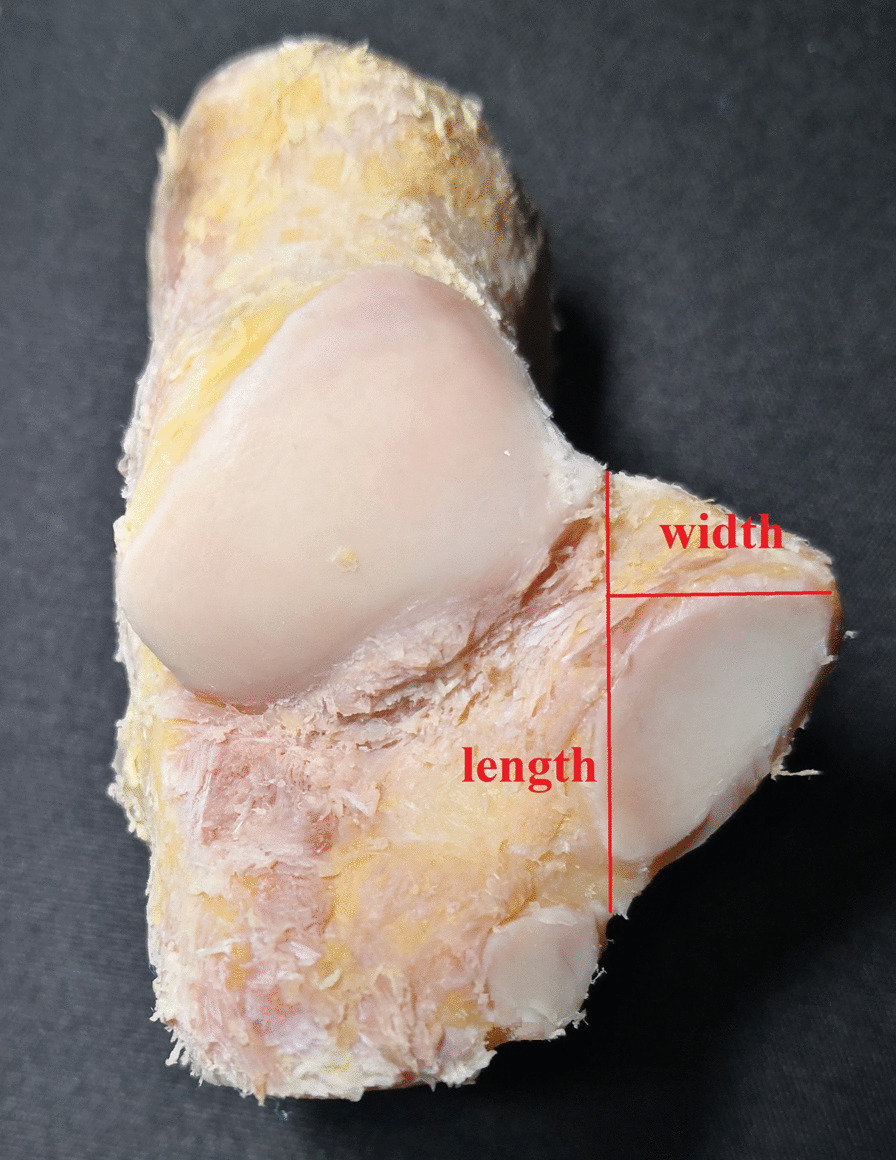
Length and width of the sustentaculum tali

**Fig. 5:**
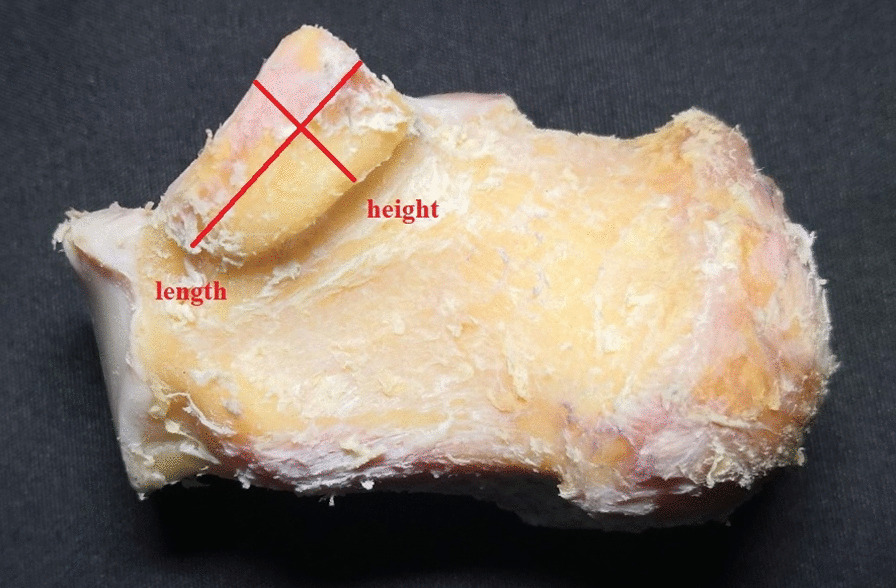
Length and height of the sustentaculum tali

The inner skin, subcutaneous tissue, fascia, blood vessels, nerves and tendons of the ankle were removed, and the anterior tibiotalar ligament, posterior tibiotalar ligament, tibiocalcaneal ligament, tibioscaphoid ligament, and talar navicular ligament were preserved. The medial part of the calcaneus, talus, medial malleolus, and navicular bone were exposed (Fig. 3). The arrangement of surrounding bones along the long axis “r” direction and the short axis “t” direction of the sustentaculum tali was observed, and the possible influence on the X-ray images of the sustentaculum tali was deduced. Photos of medial malleolar specimens were taken parallel to the plantar plane with a Sony-NET-3N50i camera made in Thailand. The obtained photos were imported into the computer and processed with PhotoScape mapping software. The lines “m” and “r” were calibrated, and the anteversion angle “α” was measured. The talus and calcaneus were retained, other tissue structures were removed, and the anatomical relationship between the talus and the sustentaculum tali was observed. According to the morphological structure of the sustentaculum tali, the length of the sustentaculum tali was defined as the maximum anteroposterior diameter of the base surface, the width was the coronal diameter of the posterior 1/3 of the basal surface and the inner edge of the sustentaculum tali, and the height was the thickness of 1/3 of the middle and posterior side of the sustentaculum tali (Figs. 4, 5). The length, width and height of each specimen sustentaculum tali were measured separately.

### Dissection of sustentaculum tali and X-ray imaging

The sustentaculum tali is located on the inside of the calcaneus and looks like an “auricle”, which is higher than the anterior of the calcaneus and extends to the inside; the base is flush and continuous with the inner wall of the calcaneus. The sagittal diameter of the sustentaculum tali is longer than that in other planes, the coronal diameter is slightly narrow, and the middle and posterior 1/3 parts are the widest. The posterior part of the sustentaculum tali is raised and gathers to form the posterior wall of the inner edge of the calcaneal groove. The anterior part is depressed and narrowed to the posterior side of the inner edge of the calcaneal protrusion. In the medial view of the ankle, taking the plantar plane as the reference plane, the sustentaculum tali body is tilted forward and downwards to form an anteversion angle (α). This anteversion angle is the axial projection angle of the sustentaculum tali, which can display the width and height of the sustentaculum tali. The obtained image is called the sustentaculum tali axial X-ray image. The only articular surface of the sustentaculum tali is the medial-posterior facet, which supports the talus and forms the medial posterior facet with the talus. The inclination direction of the medial posterior facet is consistent with the long axis of the sustentaculum tali; therefore, the short axis of the sustentaculum tali is perpendicular to the long axis and is also perpendicular to the medial posterior facet. The X-ray projection along the short axis “t” can directly reflect the length and width of the anterior of the sustentaculum tali and position of the sustentaculum tali in the frontal X-ray image. According to this principle, the frontal X-ray projection angle “β” of the sustentaculum tali can be calculated from the anteversion angle “α”.

### Placing the sustentacular screw

**Fig. 6:**
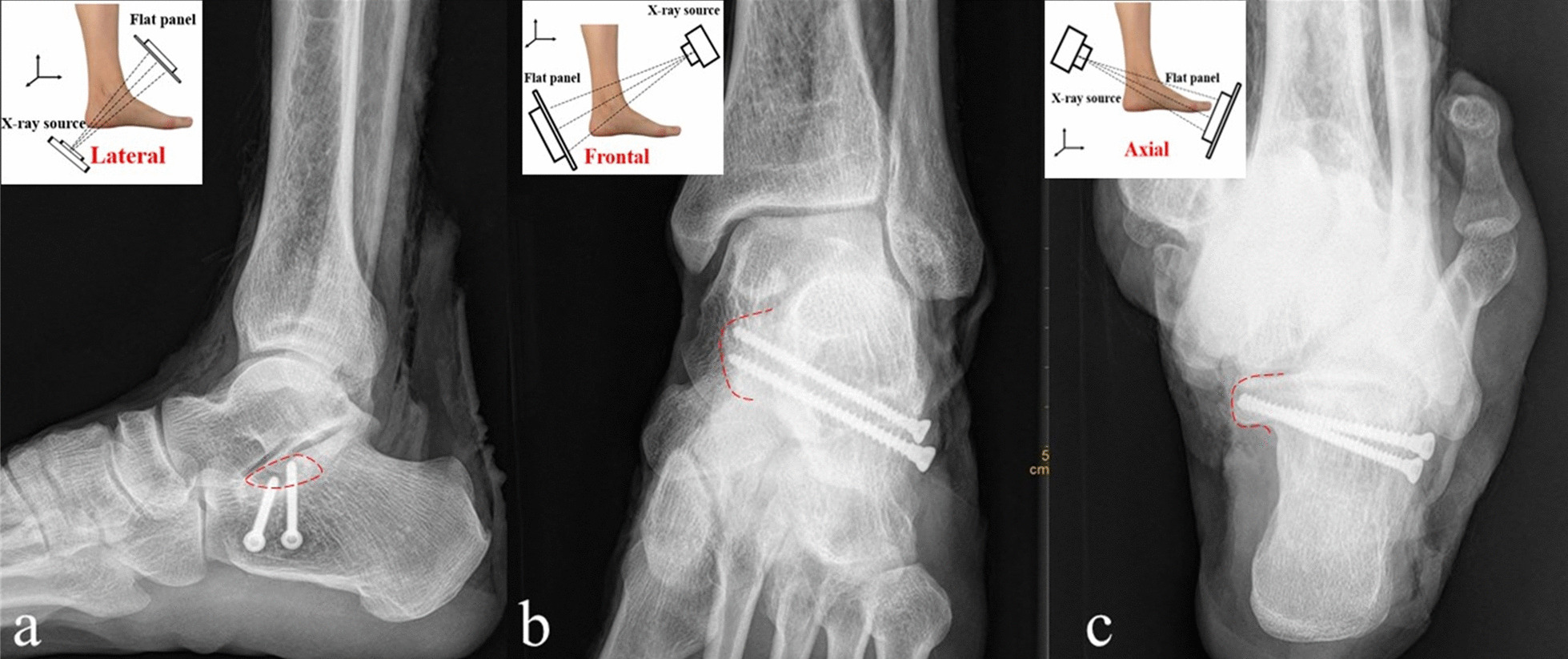
Sustentacular screw X-ray image. The red dotted line marks the outline of sustentaculum tali. **a**: Lateral X-ray of the ankle; **b**: frontal X-ray image of the sustentaculum tali; **c**: axial X-ray image of the sustentaculum tali

We randomly selected 30 specimens for in whom sustentacular screws would be placed. Screws were placed from the lateral side of the calcaneus towards the sustentaculum tali in each specimen. Under sustentaculum tali axial and frontal X-ray fluoroscopy, the direction of screw placement was dynamically adjusted. The screw placement method was as follows: The lateral wall of the anterior calcaneus was taken 5–10 mm behind the articular surface of the calcaneus cuboid as the screw insertion point, and holes were drilled in the direction of the talar process of the sustentaculum tali. The adjustment of the screw placement direction and the judgement of the screw placement depth were mainly based on fluoroscopic images. The direction of the screw can be estimated by drawing an extension line of the screw on the obtained image. If the extension line was too high or too low, raised or lowered the screw tail until the extension line was in the desired position. If the screw direction was skewed to the left or right, the adjustment method was similar. Two screws with a suitable length of 3.0 mm in diameter were inserted into the sustentaculum tali from the lateral wall of the anterior part of the calcaneus, and screw placement was observed (Fig. 6).

### Statistical analysis

Statistical data were processed with SPSS 23.0 statistical software and a threshold of *p* < 0.05 indicated statistical significance. The results, such as the length, width, height and anteversion angle of the sustentaculum tali, are reported as the mean ± SD (X ± S). The measurement differences between the specimens in the dissection and imaging groups were assessed for significance using independent *t* tests after confirming that the data followed a normal distribution.

## Result

### The position relationship between the sustentaculum tali and adjacent bones

**Fig. 7:**
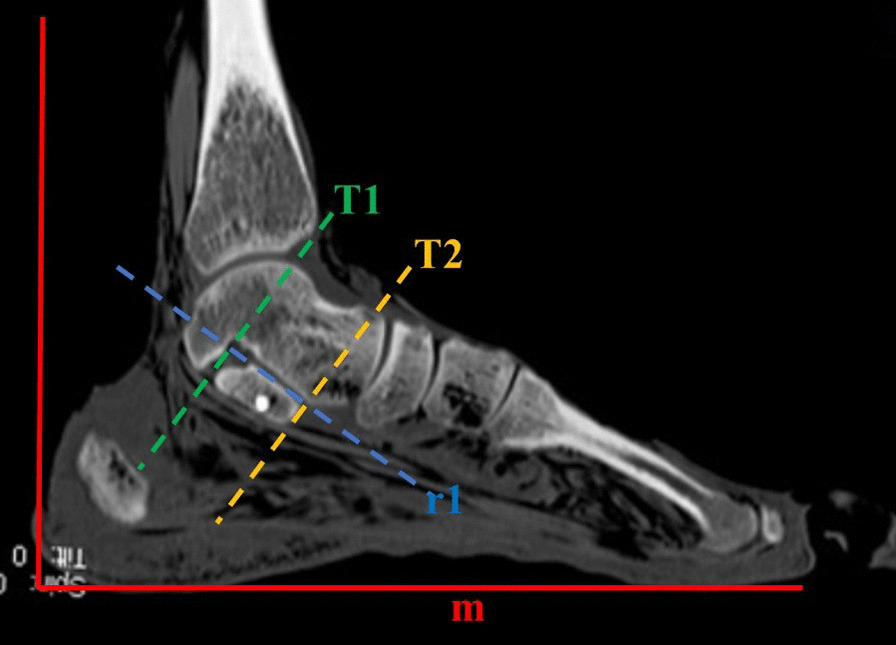
Sagittal CT image of the ankle joint. (r1) Upper surface of the sustentaculum tali; (T1) posterior edge of the sustentaculum tali; (T2) anterior edge of the sustentaculum tali

The sustentaculum tali is located approximately 25 mm below the medial malleolus, and the two are separated by the talus and do not constitute a joint. The tangent line “r1” on the upper surface of the sustentaculum tali is located below the medial malleolus, which indicates that the medial malleolus does not affect the appearance of the sustentaculum tali on axial X-ray images (Fig. 7). Similarly, the posterior edge of sustentaculum tali “T1” does not pass through the medial malleolus, indicating that the medial malleolus and sustentaculum tali do not overlap in the frontal direction; that is, it does not affect the appearance of the sustentaculum tali on frontal X-ray images. The talus is located above the sustentaculum tali and constitutes the medial posterior joint. In the medial view of the ankle, the sustentaculum tali is surrounded by the talus in the posterior, upper, and anterior directions. When in the frontal direction, the sustentaculum tali is partially obscured by the talus, which affects the appearance of the sustentaculum tali on frontal X-ray images. In the axial direction, the sustentaculum tali overlaps with the posterior process of the talus, which affects the appearance of the sustentaculum tali on axial X-ray images. However, the posterior process of the talus is thin and has less bone, which has little effect on imaging findings. In the frontal direction, the navicular bone is located in front of the sustentaculum tali anterior surface “T2” and does not affect the appearance of the sustentaculum tali on frontal X-ray images. In the axial direction, the navicular bone is located in front and above the sustentaculum tali and does not block the sustentaculum tali on axial X-ray images.

### Sustentaculum tali measurement data

A total of 42 foot and ankle specimens were measured in this study. The data of specimens in the anatomy group were measured by two anatomy professors using an electronic ruler with an accuracy of 0.01 mm, and the data of specimens in the image group were measured by a professional imaging doctor and an orthopaedic doctor through 3D CT scan images. All personnel received formal training, the recorded data passed the consistency test, and the ICC value was more than 0.75. The final data consisted of the average of two measurements (Table 1). There was no significant difference between specimens in anatomy and imaging groups in the sustentaculum tali length (23.96 ± 1.66 vs. 23.75 ± 1.91), width (14.98 ± 1.83 vs. 15.22 ± 1.22), height (10.97 ± 1.07 vs. 10.71 ± 1.32), or anteversion angle (30.81 ± 2.21 vs. 30.68 ± 2.86).

**Table 1:** Sustentaculum tali measurement data

Group	Length(mm)	Width(mm)	Height(mm)	Anteversion angle(°)
Anatomy group	23.96 ± 1.66	14.98 ± 1.83	10.97 ± 1.07	30.81 ± 2.21
Inage group	23.75 ± 1.91	15.22 ± 1.22	10.71 ± 1.32	30.68 ± 2.86
*P*	0.197	0.087	0.119	0.777

### Specimen placing screw results

**Fig. 8:**
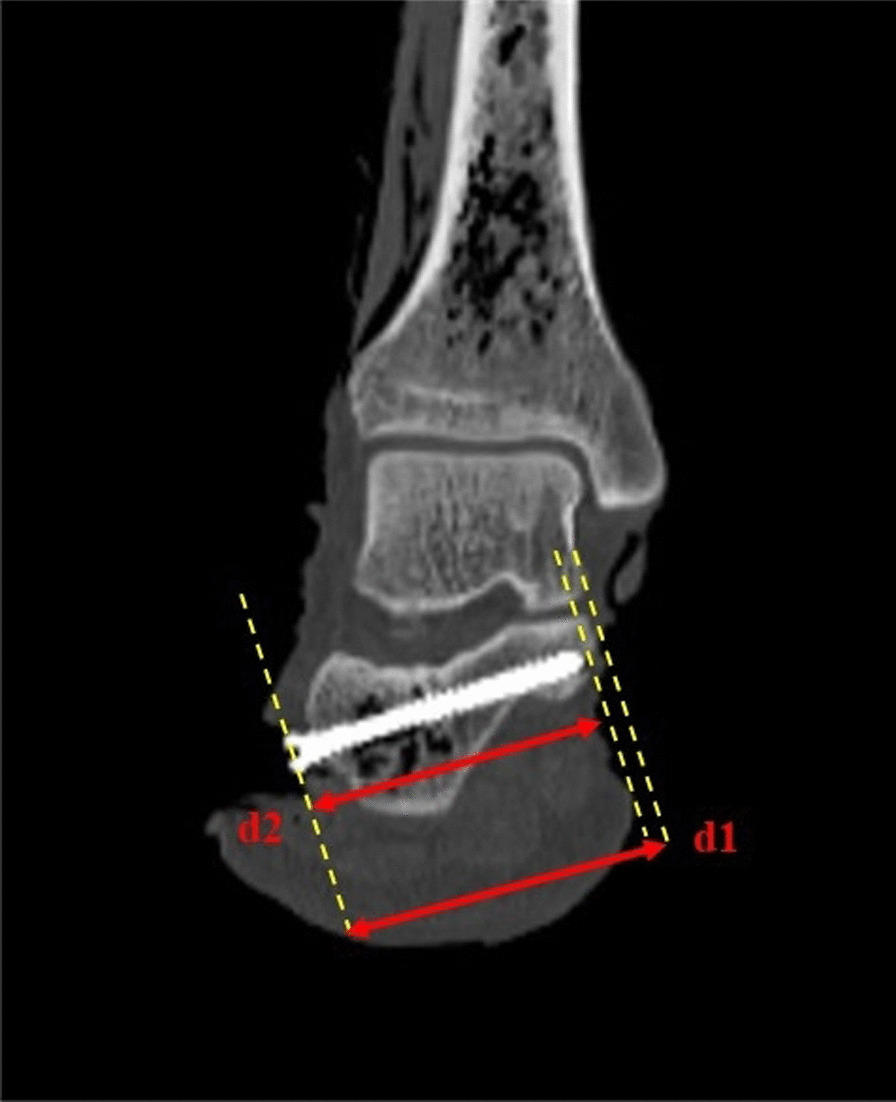
Oblique coronal CT image of ankle joint. (d1) Maximum length through the central axis of the screw; (d2) length of the screw

MSCT scans were performed on all the screwed specimens to observe the screw position. The length of the screw (d2) and the maximum length through the central axis of the screw (d1) were measured in the oblique coronal plane (Fig. 8). When 0 < d1–d2 < 10 mm and the screw was located in the distance-carrying process, it was considered that the screw is placed well. A total of 60 screws were placed in the 30 specimens; of these, 54 screws were in good position, and the screw placement rate was 90% (54/60). In our study, we found that one screw penetrated the cortical bone in two specimens, and 4 screws did not enter the sustentaculum tali. These screws were all the second screws placed in each specimen. This finding related to the interruption of the views of the first screw that was placed during the fluoroscopy progress. A total of 10% (6/60) of the screws were not placed in a satisfactory position.

Axial X-ray images of the sustentaculum tali can be obtained at the Harris heel, 30° relative to the axis of the foot. Images perpendicular to this projection angle are needed to locate the sustentaculum tali in addition to frontal X-ray images. The position and shape of the sustentaculum tali can be clearly observed, and it is seldom affected by adjacent bones in the obtained images. A total of 60 screws were placed in 30 specimens under this fluoroscopic technique, with an accuracy rate of 90%.

## Discussion

The main contribution of this research was to define and measure the length, width and height of the sustentaculum tali. The concept and method of axial and frontal X-ray imaging of the sustentaculum tali was described for the first time, and its feasibility and reliability were verified by specimen experiments. The shape and location of the sustentaculum tali can be clearly shown in axial and frontal X-ray images of the sustentaculum tali, which can help the surgeon to determine the positional relationship between the screw and the sustentaculum tali. This study solves the problem of an "obscured" sustentaculum tali for screw fixation during calcaneal fracture surgery and is expected to improve the accuracy of screw placement in the sustentaculum tali.

As an important supporting structure on the medial side of the calcaneus, the sustentaculum tali is usually referred to as a “constant” fragment, which has important reference value in calcaneal fracture surgery [14–16]. It was reported that in calcaneal fractures, sustentacular fractures accounted for approximately 44%, and only 10% of them were displaced [17]. Therefore, the sustentaculum tali is often used as a scaffold to reduce other fracture fragments and restore the congruity of the subtalar joint in calcaneal fracture surgery. Anatomical reconstruction of displaced intra-articular calcaneal fractures contributes to reducing the incidence of long-term complications, such as arthritis of the subtalar joint, tendinitis and persistent medial malleolar pain [10, 18, 19]. Some scholars [13, 20] have developed devices that can provide guidance and can increase the success rate of sustentacular screw insertion, but not all hospitals are equipped with such devices. In contrast, the axial and frontal X-ray imaging method we developed to visualise the sustentaculum tali is easier to learn and promote than other options.

Axial and frontal X-ray imaging of the sustentaculum tali is based on the anatomical and imaging measurement data of the foot and ankle specimens and was designed according to the principle of "less bone occlusion, good projection angle, and accurate entry point" to obtain high-quality X-ray images of the sustentaculum tali. This fluoroscopy method provides clearer and more accurate views than the calcaneal axis for the observation of the sustentaculum tali. Combined with the recovery of the Gissane and Bohler angles observed by the lateral X-ray image of the calcaneus during the operation, we determined whether the fracture reduction and the screw position were appropriate.

Gitajn [21] evaluated the ability of the Harris heel view to allows surgeons to observe sustentacular screw placement during calcaneal fracture surgery. His study shows that the Harris heel view, previously described as the beam angled 35° to 45° relative to the axis of the foot, cannot accurately confirm the actual placement of the sustentacular screw. He believes that 2 axial heel views obtained at approximately 10°–20° and 20°–50° can confirm accurate screw placement in the sustentaculum. His method is to observe 5 different screw placements (screws placed anatomically within the sustentaculum, inferior, superior, anterior and posterior to the centre of the sustentaculum) through 5 different angles (10-, 20-, 30-, 40-, and 50-degree Harris heel views) and evaluate which viewing angle is more accurate. Our method is completely different from his. Our view angle was obtained through anatomical and imaging measurements, and we observed sustentacular screw placement in axial and frontal views. Our research provides an accurate projection angle of the C-arm in calcaneal fracture surgery, and doctors do not need to rely on experience or repeated fluoroscopy to obtain the desired image.

To obtain axial and frontal views of the sustentaculum tali during the operation, the position of the rotating part of the C-arm must be determined according to the patient's body position. When the patient lies in the supine position, the rotating arm is placed vertically, and adjustment is relatively simple. When the patient lies on the side, the rotating arm needs to be placed horizontally. However, whether the patient is in the supine position or the lateral position, fluoroscopy of the ankle joint needs to be performed in a functional position, and the plantar plane is used as the reference measurement surface. During the operation, the ankle can be fixed in a functional position with a sterile bandage; one end is pulled to the forefoot, and the other end is fixed to the upper end of the calf. The projection of the long axis and short axis of the sustentaculum tali was marked on the body surface before fluoroscopy. When the centerline of the C-arm tube X-ray transmitter and receiver coincides with the long axis, the axial X-ray image of the sustentaculum tali is obtained. Similarly, frontal imaging can also be obtained. To obtain high-quality images in the first imaging session, we recommend measuring the anteversion angle through CT images of the normal ankle before surgery to determine a more accurate projection angle.

There were several limitations in our study. All specimens in this study were from Asian adults, and the measured data may have regional limitations. The new X-ray fluoroscopy techniques of sustentaculum tali reported in this study need to be further verified and improved in the clinic.

In summary, this study introduced a new fluoroscopy technique for sustentaculum tali based on anatomy and imaging. This fluoroscopy method is simple to perform and can clearly display the position and shape of the sustentaculum tali, which is expected to improve the accuracy of sustentacular screw placement.

## Data Availability

All data generated or analyzed during this study are included in this published article.
